# K_V_7/KCNQ Channels Are Functionally Expressed in Oligodendrocyte Progenitor Cells

**DOI:** 10.1371/journal.pone.0021792

**Published:** 2011-07-05

**Authors:** Wei Wang, Xiao-Fei Gao, Lin Xiao, Zheng-Hua Xiang, Cheng He

**Affiliations:** Institute of Neuroscience and Key Laboratory of Molecular Neurobiology of the Ministry of Education, Neuroscience Research Center of Changzheng Hospital, Second Military Medical University, Shanghai, China; Louisiana State University Health Sciences Center, United States of America

## Abstract

**Background:**

K_V_7/KCNQ channels are widely expressed in neurons and they have multiple important functions, including control of excitability, spike afterpotentials, adaptation, and theta resonance. Mutations in KCNQ genes have been demonstrated to associate with human neurological pathologies. However, little is known about whether K_V_7/KCNQ channels are expressed in oligodendrocyte lineage cells (OLCs) and what their functions in OLCs.

**Methods and Findings:**

In this study, we characterized K_V_7/KCNQ channels expression in rat primary cultured OLCs by RT-PCR, immunostaining and electrophysiology. KCNQ2-5 mRNAs existed in all three developmental stages of rat primary cultured OLCs. K_V_7/KCNQ proteins were also detected in oligodendrocyte progenitor cells (OPCs, early developmental stages of OLCs) of rat primary cultures and cortex slices. Voltage-clamp recording revealed that the I_M_ antagonist XE991 significantly reduced K_V_7/KCNQ channel current (I_K(Q)_) in OPCs but not in differentiated oligodendrocytes. In addition, inhibition of K_V_7/KCNQ channels promoted OPCs motility in vitro.

**Conclusions:**

These findings showed that K_V_7/KCNQ channels were functionally expressed in rat primary cultured OLCs and might play an important role in OPCs functioning in physiological or pathological conditions.

## Introduction

The KCNQ gene family encodes five voltage-gated delayed rectifier K^+^ channels K_V_7.1-5, and four of these K_V_7.2-5 are expressed in the nervous system [1], [2]. There they form subunits of voltage-gated K^+^ channel originally termed the ‘M-channel’ and the current called M current, which has been demonstrated to assist in stabilizing the membrane potential in the presence of depolarizing currents and contributing to the resting potential of neurons [3], [4]. In CNS, K_V_7 channels form through homo- or heteromeric assembly of K_V_7.2 to K_V_7.5 subunits. So far, homomeric compositions are shown for K_V_7.2-5 subunits; heteromeric compositions are represented by K_V_7.2+3, K_V_7.3+4 and K_V_7.3+5 channels [2]. In most neurons native K_V_7 channels are composed of K_V_7.2 and K_V_7.3 subunits [5] or sometimes of homomeric K_V_7.2 subunits [6], [7], although probably with a contribution by K_V_7.5 subunits in some neurons [8]; K_V_7.4 subunits are predominantly expressed in the auditory and vestibular systems, but also probably contribute to K_V_7 channels in central dopaminergic neurons [9]. Recent evidences suggest that K_V_7 channels have profound effects on neuronal excitability [10]–[15]. Inhibition of channel activity, by either a blocking drug such as linopirdine (DuP 996) [16] or 10, 10-bis(4-pyridinyl- methyl)-9(10 H)-anthracenone (XE991), or expression of a dominant-negative K_V_7.2 construct, strongly enhances repetitive firing and even effects postnatal brain development [17]. Their mutations have been associated with human neurological pathologies including auditory diseases [1], [2]. Mutations in either K_V_7.2 or K_V_7.3 lead to benign familial neonatal seizures [18] as do mutations in K_V_7.5 [19], [20]. In addition, mutations in K_V_7.4 are associated with progressive hearing loss [21]–[23].

Oligodendrocytes are generated from oligodendroglial progenitor cells (OPCs) which proliferate in the subventricular zone and migrate to formative white matter regions, where they further proliferate, differentiate, and form myelin sheaths around axons [24], [25]. Migration of OPCs is an essential step not only during the early stage of oligodendrocyte lineage cells (OLCs) development but also in some demyelination pathological conditions such as Multiple Sclerosis (MS) and other variety of CNS injuries [26]–[28]. Several ion channels have been identified recently in OLCs to participate in regulation of OPCs migration including K_V_ 3.1[29], voltage gated Ca^2+^ channel [30], [31] P2X7 receptor [32], GABA receptor [33], glutamate (AMPA and/or kainate) receptor [34] etc. In addition, previous studies indicated that the various K^+^ channels were linked to cell migration. Kv7.1 has been reported to regulate invasiveness of stem-like cell types [35]. Activation of K_V_ channel promotes migration of intestinal epithelial cells [36]. K_V_10.1 is involved in adhesion and viability of CHO cells [37]. K_V_11.1 participates in tumor cells invasion [38] and inhibition of K_V_1.3 suppresses the motility and activation of effector memory T (Tem) cells [39]. OLCs express all six members of the delayed rectifier Shaker family K^+^ channels, Kv1.1–Kv1.6 [40]–[45], inwardly rectifying K^+^ (K_ir_) channels Kir2.1, Kir1.1 and Kir4.1 [46], [47] and Kv3.1[29]. However, whether OLCs functionally express K_V_7 channels is still unknown. In this paper, we studied the expression and function of K_V_7 channels in OLCs.

## Results

### The mRNAs of K_V_7.2–5/ KCNQ2-5 were detected in rat primary cultured OLCs

**Figure 1:**
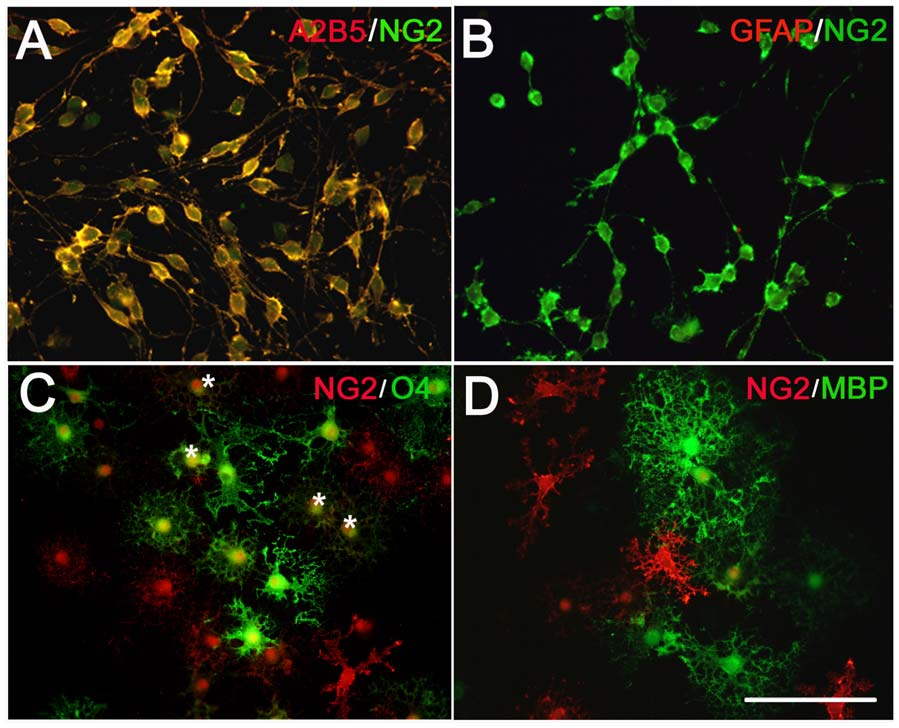
Morphological and immunostaining characterization of OLCs in rat primary cultures. (A) Double immunostaining of cultured OPCs showing that A2B5-positive cells (red) were also immunopositive for anti-NG2 (green). (B) OPCs (NG2-positive, green) were negative for anti-GFAP (red). (C) There were NG2-positive cells (red) after differentiation in T3 contained medium for two days. Immature oligodendrocytes (IOs) were stained with O4 antibody (green), a marker of IOs. Some cells co-localized with O4 and NG2 (Asterisk) (D) Four days later, NG2-positive cells (red) still existed and mature oligodendrocytes (MOs) were stained with MBP antibody (green), a marker of MOs. Scar bar = 100 µm.

**Figure 2:**
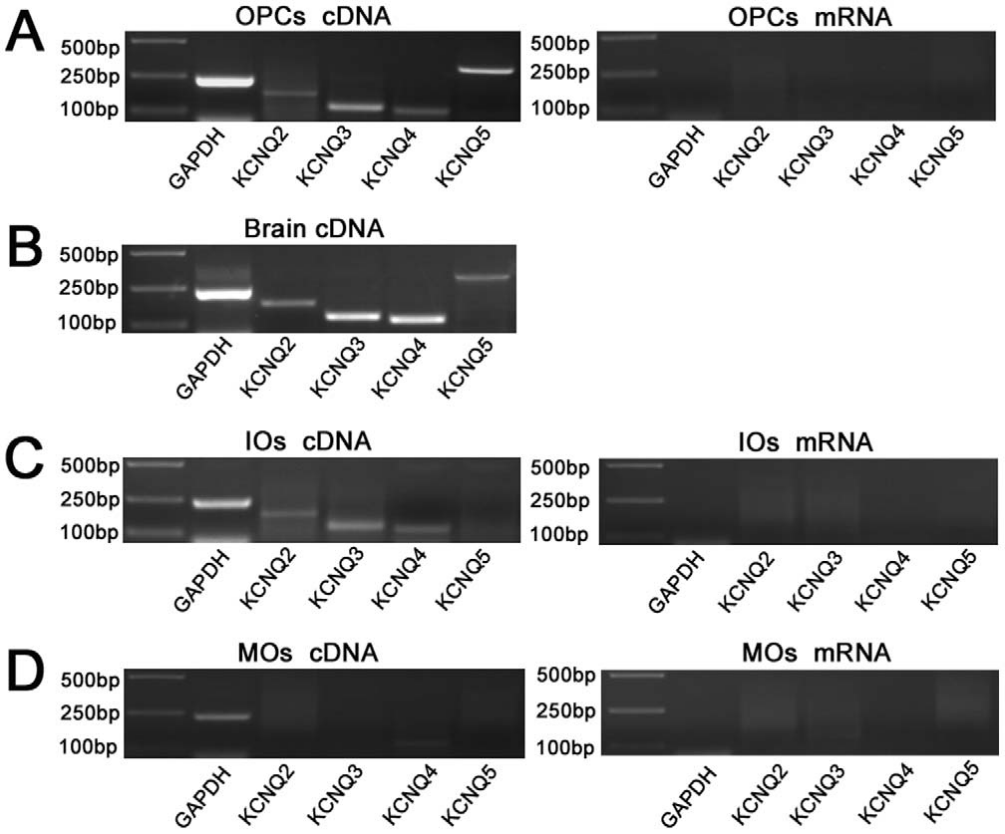
RT-PCR analysis for primary cultured OLCs. (A–D, left) The mRNA of GAPDH (243 bp), KCNQ2 (172 bp), KCNQ3 (121 bp), KCNQ4 (110 bp) and KCNQ5 (320 bp) was identified in OLCs and whole forebrain (positive control) by RT-PCR. (A, C, D, right) RNA from OLCs without reverse transcription was performed PCR procedure directly as negative control.

Immunocytochemical markers allow for the distinction of three consecutive phenotypically defined stages of OLCs development in vitro: the bipolar GFAP^−^A2B5^+^NG2^+^ OPCs, multipolar O4^+^GalC^−^ IOs, and complex process bearing MBP^+^GalC^+^ MOs [48], [49]. In the present study, we got highly pure GFAP^−^A2B5^+^NG2^+^ OPC cultures (98.8±0.2%, assessed by immunocytochemical staining) (Fig. 1 A, B). In differentiation medium, OPCs developed into O4^+^ IOs and MBP^+^ MOs (Fig. 1 C, D). As KCNQ1 was not detected in neural system [1], [2], we examined the mRNAs of KCNQ2-5 in cultured OLCs by RT-PCR. We found that KCNQ2-5 mRNA were all present in cultured OPCs (Fig. 2A left). KCNQ5 was undetectable in IOs (Fig. 2C left). In MOs, only KCNQ4 was detectable very weakly (Fig. 2D left). No positive line was present in negative control which implied that the mRNA was not contaminated with genome DNA (Fig. 2A, C, D right).

### Localization of K_V_7.2-5/ KCNQ2-5 in OLCs

**Figure 3:**
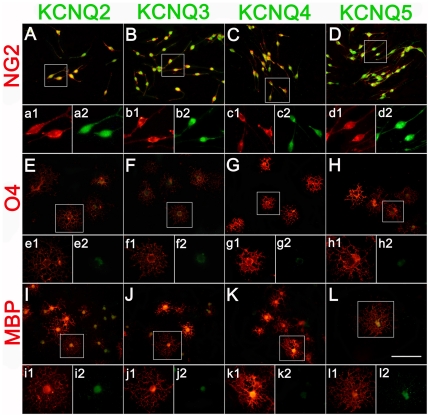
Immunofluorescence localization of K_V_7.2-5/KCNQ2-5 subunits on the OLCs in rat primary cultures. (A–D) Co-localization of immunostaining for K_V_7.2-5 subunits (green) and OPCs (NG2-positive, red) is displayed in the merged image. Higher magnification of the boxed area in A, B, C, D was shown in a1, a2; b1,b2; c1,c2; d1,d2. (E–H) The expression of K_V_7.2-5 subunits (green) on IOs (O4-positive, red). Higher magnification of the boxed area in E, F, G, H was shown in e1, e2; f1,f2; g1,g2; h1,h2. (I–L) The expression of K_V_7.2-5 subunits (green) on MOs (MBP-positive, red). Higher magnification of the boxed area in I, J, K, L was shown in i1, i2; j1,j2; k1,k2; l1,l2. Scar bar = 100 µm.

**Figure 4:**
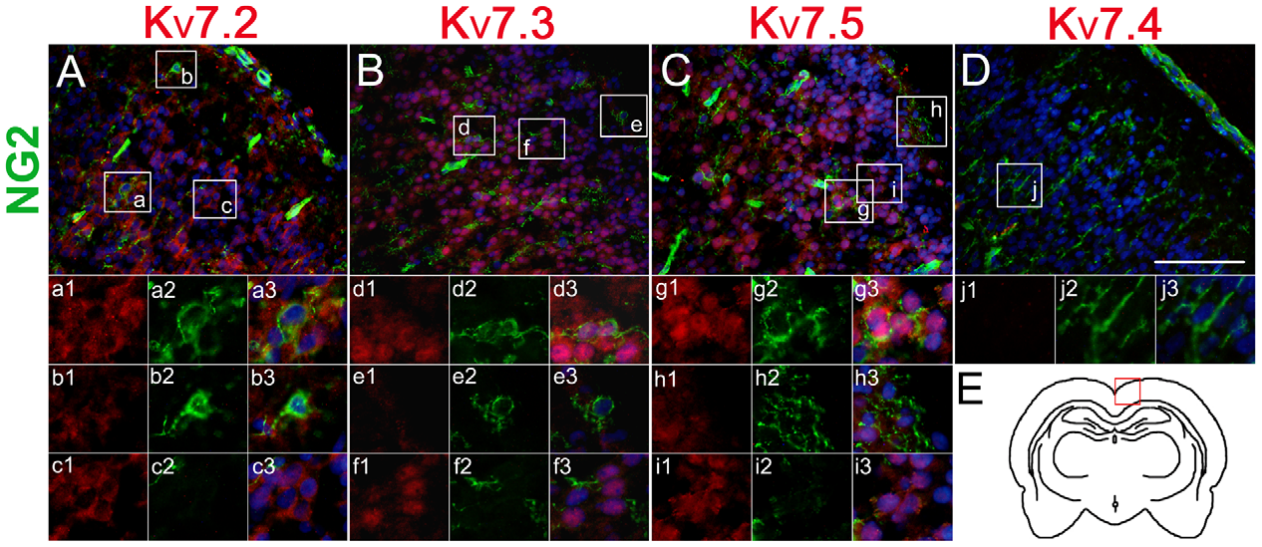
Immunofluorescence localization of K_V_7.2-5/KCNQ2-5 on NG2-positive cells (green) of the rat brain slices. (A–C) Co-localization of K_V_7.2, 3, 5 and OPCs is displayed in the merged image. Higher magnification of the boxed area in A, B and C was shown in a1–a3; b1–b3;c1–c3;d1–d3;e1–e3;f1–f3;g1–g3;h1–h3 and i1–i3. (D) K_V_7.4 was not detected on OPCs. j1–j3: higher magnification of the boxed area in D. The nuclei were stained with Hoechst (blue). (E) Schematic diagram of the brain coronal and the box represented A–D areas. Scar bar = 100 µm.

The expression of K_V_7.2-5 in cultured OLCs was further confirmed by immunostaining. The antibodies of anti-NG2, anti-O4 and anti-MBP were used to identify OPCs, IOs, and MOs in cultures respectively. The staining for K_V_7.2-5 was on the soma and processes of OPCs. The immunofluorescence signals were positive in both the cytoplasm and the cell membrane of OPCs (Fig. 3A–D). With the maturation of OLCs, the immunofluorescence signals of K_V_7.2-5 became weaker and were restricted to the cell bodies in IOs, and MOs. (Fig. 3E–H, I–L). Immunohistochemistry was also performed to verify the expression of K_V_7.2-5 proteins in OPCs in vivo. In cortex, K_V_7.2, 3 or 5 were detected to localize on a part of NG2^+^ OPCs (26±9%, 27.1±11% and 30.5±7% respectively) (Fig. 4A–C, a1–a3; d1–d3; g1–g3), while other part of NG2^+^ OPCs did not express K_V_7.2, 3 or 5 (Fig. 4A–C, b1–b3; e1–e3; h1–h3). We also found that some cells, which expressed K_V_7.2, 3 or 5, were not NG2 positive (Fig. 4A–C, c1–c3; f1–f3; i1–i3). Those cells may be neurons or other glia cells. All NG2^+^ cells were K_V_7.4 negative (Fig. 4D, j1–j3).

### K_V_7/KCNQ Channel currents (I_K(Q)_) in OLCs

**Figure 5:**
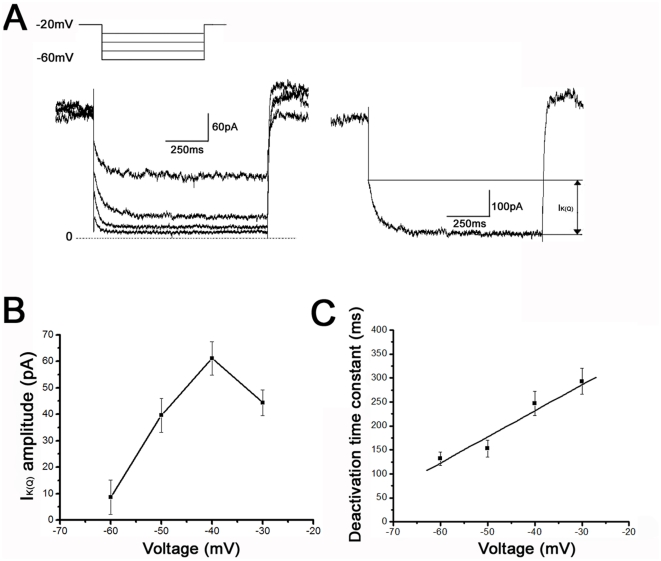
K_V_7/KCNQ channel current (I_K(Q)_) in OPCs of rat primary cultures. (A) I_K(Q)_ was measured with whole cell patch clamp recording from OPCs. Left insert: Standard I_M_ deactivation voltage protocol used to measure I_K(Q)_. Hyperpolarizing voltage steps were given from a holding potential of −20 to −60 mV (in 10 mV decrements). Currents recorded are shown below; the dashed line represents the zero current level. Right: Current recorded in response to the voltage step to −40 mV. I_K(Q)_ was measured as the inward relaxation current caused by deactivation of I_K(Q)_ during the voltage step; i.e., the difference between the instantaneous current at the beginning and the steady-state current at the end of the voltage step (arrows). (B) Current–voltage relationship for I_K(Q)_ (mean data from 17 OPCs) showing that I_K(Q)_ amplitude was voltage dependent and was largest at −40 mV. (C) I_K(Q)_ deactivation time constants were directly related to voltage (mean data from 17 OPCs). Correlation coefficient r = 0.93.

**Figure 6:**
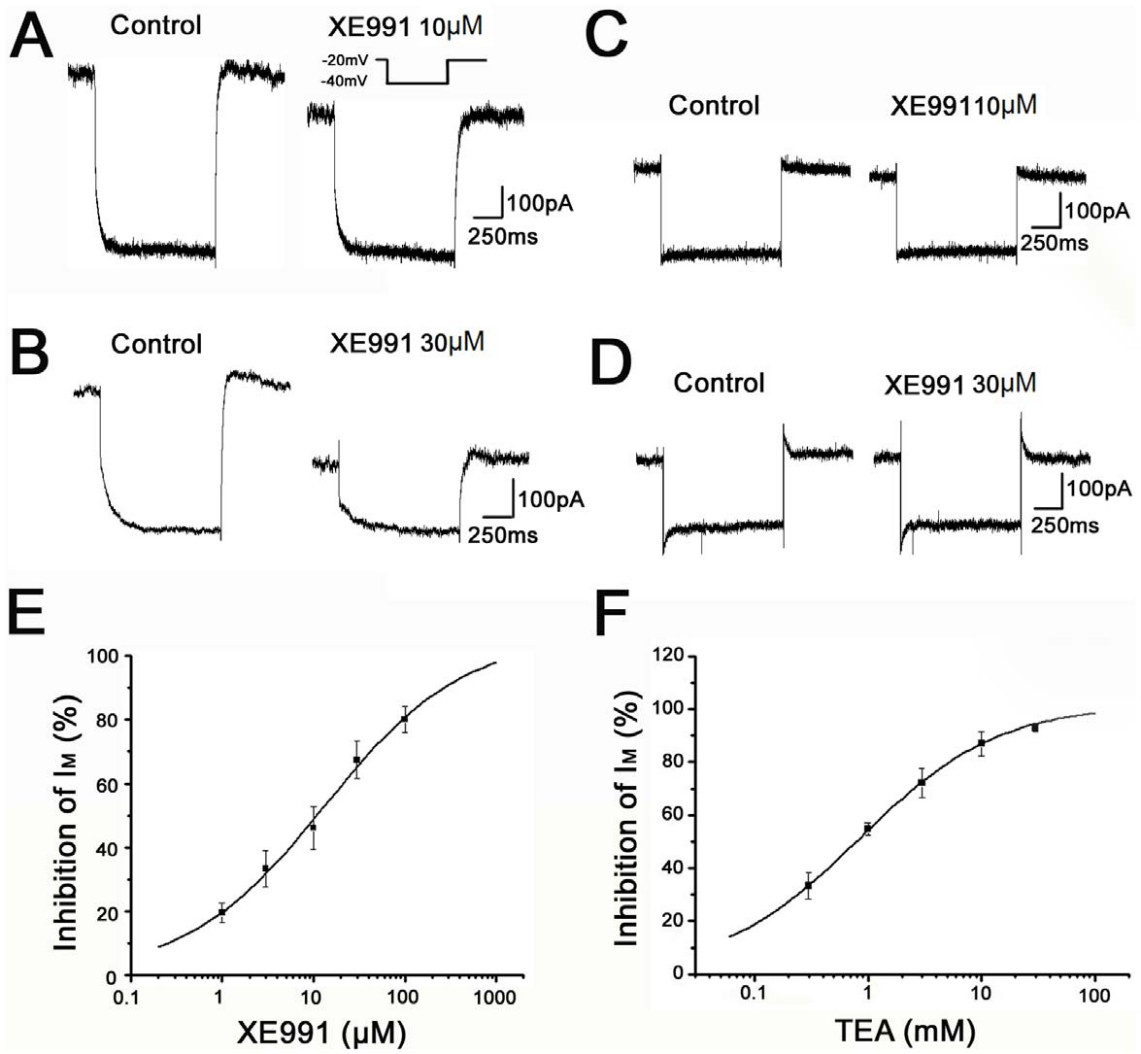
Inhibition of I_K(Q)_ by XE991 and TEA. (A, B) Representative current traces, which were recorded before (Control) and after extracellular application of XE991 (10 µM, 30 µM) in OPCs. (C, D) Representative current traces, which were recorded before (Control) and after extracellular application of XE991 (10 µM, 30 µM) in differentiated oligodendrocytes. Insert: 1-s-long hyperpolarizing voltage step from a holding potential of −20 to −40 mV was given to monitor I_K(Q)_. (E) Concentration–response curve showing the mean percentage inhibition of I_K(Q)_ amplitude as a function of the log XE991 concentration for 54 OPCs. Smooth curve was fit with the Hill equation. IC50 value for XE991 inhibition determined from this pooled log concentration–response curve was 13.3 µM. n = 0.63, R^2^ = 0.99. (F) Concentration–response curve showing the mean percentage inhibition of I_K(Q)_ amplitude as a function of the log TEA concentration for 20 OPCs. Smooth curve was fit with the Hill equation. IC50 value for TEA inhibition determined from this pooled log concentration–response curve was 0.84 mM. n = 0.7, R^2^ = 0.99.

In order to determine the K_V_7 channels are functionally expressed in OLCs, we recorded 61 cultured OLCs with whole-cell patch clamp recording to evaluate the electrophysiological property of the currents. In an attempt to isolate the K_V_7 channel currents from other voltage-gated K^+^ currents, the membrane potential was held at a relatively depolarized potential (−20 mV) to activate K_V_7 channels [3], [50] and to inactivate many of the other K^+^ channels that activate in this membrane potential region [51]–[53]. The membrane potential was then stepped down to more hyperpolarized potentials (−60 mV, in 10 mV decrements) for 1 s to deactivate the K_V_7 channels (Fig. 5A). The amplitude of the I_K(Q)_ was measured as the difference between the instantaneous current at the onset of hyperpolarization and the steady-state current at the end of voltage command [3] (Fig. 5A, right). Fig. 5B shows the current–voltage relationship of I_K(Q)_ from 17 OPCs. The mean I_K(Q)_ amplitude was voltage dependent and the maximal I_K(Q)_ amplitude (61.16±6.32 pA) was measured at −40 mV. The deactivation time constant of I_K(Q)_ was determined by fitting the current curves measured at each voltage with a single exponential function. Fig. 5C shows the mean deactivation time constant of I_K(Q)_ as a function of voltage (n = 17). The mean I_K(Q)_ deactivation time constant was 292.94±26.79 ms (−30 mV), 246.94±24.97 ms (−40 mV), 152.85±17.59 ms (−50 mV) and 131.89±14.18 ms (−60 mV), indicating that it was voltage dependent. Note that the deactivation time constant was a linear function of voltage (correlation coefficient r = 0.93) and was shorter at more negative membrane potentials which means at these potentials, I_K(Q)_ was deactivated faster. We also recorded I_K(Q)_ in IOs and MOs and the inward deactivation relaxation currents were almost not existed. (Fig. 6C, D). Consequently, in this study, the characterizations of I_K(Q)_ mainly were obtained from OPCs.

XE991 has been shown to be a potent and selective inhibitor for M current (I_M_) in native neurons and currents from artificial expressed K_V_7 channels [5] and has little impact on Kv2.1 [54]. In our experiments, I_K(Q)_ was monitored with a 1-s-long hyperpolarizing voltage stepped from a holding potential of −20 to −40 mV. XE991 (10 µM) reduced about 46.1±6.8% I_K(Q)_ in OPCs. Higher concentration of XE991 (30 µM) inhibited I_K(Q)_ more than a half (by 67.4±5.6%) (Fig. 6 A, B). Fig. 6E shows the pooled concentration–response curve which plots the mean percentage inhibition of I_K(Q)_ amplitude versus the log concentration of XE991 from 54 OPCs. The mean inhibition of I_K(Q)_ by XE991 was 19.5±3.1% (at 1 µM), 33.3±5.7% (at 3 µM) and 80.1±4.2% (at 100 µM). The mean data was fitted with the Hill equation (see METHODS). The IC50 for XE991 was 13.3 µM and the power term n (Hill slope), which is related to the steepness of curve was 0.63. The goodness of fit R^2^ was 0.99. In contrast, the currents in IOs and MOs were very insensitive to XE991 (Fig. 6 C, D).

Previous studies reported that different K_V_7 channel proteins have different sensitivities to TEA [55]; [56]; [5], and therefore it was of interest to examine the TEA sensitivity of I_K(Q)_ in OPCs. The voltage protocol used to measure I_K(Q)_ is the same as XE991 on I_K(Q)_ recorded in OLCs. We then applied TEA at concentrations ranging from 0.3 mM to 30 mM. TEA caused a concentration dependent reduction in I_K(Q)_ in OPCs. Fig. 6F shows the pooled concentration–response curve which plots the mean percentage inhibition of I_K(Q)_ amplitude versus the log concentration of TEA from 20 OPCs. The mean inhibition of I_K(Q)_ by TEA was 33.3%±5.1% (0.3 mM), 54.7%±2.4% (1 mM), 72.1%±5.5% (3 mM), 87%±4.6% (10 mM) and 92.8%±1.2% (30 mM). Application of 30 mM TEA completely abolished the current. The mean data was fitted with the Hill equation (see METHODS). The IC50 for TEA was 0.84 mM and the power term n (Hill slope) was 0.7. The goodness of fit R^2^ was 0.99.

### The inhibition of K_V_7/KCNQ channels promotes OPCs motility in *vitro*

**Figure 7:**
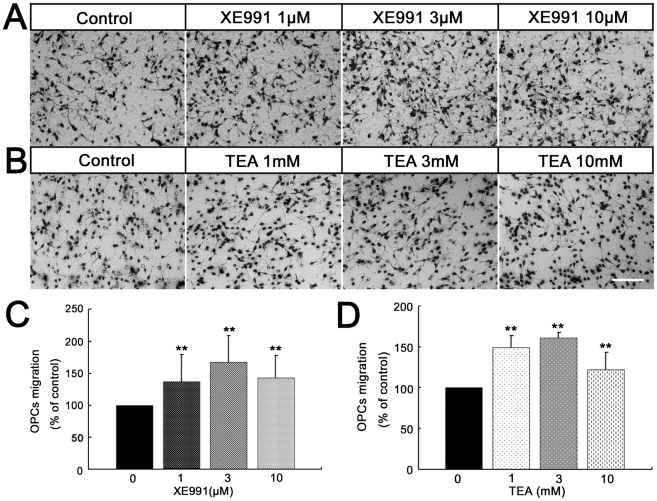
The inhibition of K_V_7/KCNQ channels promotes OPCs motility in vitro. (A,B) Photomicrograph of OPCs transmigrated through the filter in the absence or presence of XE991 or TEA. Scar bar  = 150 µm. (C, D) Quantitative assessment of migrated cells under different conditions. n(XE991) = 9; n(TEA) = 9. **P<0.01 versus control.

Besides the electrophysiological properties of the K_V_7 channels, we also investigated the effect of these channels on OPCs migration, which is important for myelin development. We measured the mobility of OPCs cultured in a Boyden chamber. After 8 h incubation with 1 µM, 3 µM or 10 µM XE991 in the lower wells of the chemotaxis chambers, the number of migrated OPCs was significantly increased compared with the control group (Fig. 7A, C), suggesting that inhibition of Kv7/KCNQ channels promotes OPCs migration. We also tested the effect of another blocker TEA on OPCs migration. As shown in Figure 7 B and D, in the presence of TEA (1 mM, 3 mM or 10 mM) the number of OPCs migrating through the transwell was significantly increased.

## Discussion

### OLCs express K_V_7/KCNQ channels

Neuronal K_V_7 channels are constructed from a family of at least four subunits (K_V_7.2–5) [1], [2], [5]. These subunits are expressed widely in the brain and prominently localized in several types of neurons [57], [58]. Some studies also suggested that a population of glial cells in the white matter expressed the K_V_7.4/5, but they didn't state clearly the type of glial cells [8], [59]. The present study represents the first attempt to identify the K_V_7 channel subunits in OLCs. The mRNA of the four genes (KCNQ2-5) was detectable in OPCs. KCNQ3 and KCNQ5 mRNAs were detected strongly, and lesser abundances of mRNAs encoding KCNQ2 and KCNQ4 were observed. KCNQ2-4 mRNAs also existed in IOs with similar expression levels. In MOs, we only find very week sign of KCNQ4 mRNA. These indicated that the transcripts of KCNQ2-5 might be down regulated during the maturation of OLCs. Previous studies found that KCNQ2, 4, 5 genes have alternative splice variants [60]-[63]. However, the primers used in this study were designed based on regions outside the putative splice variation position of rat KCNQ2-5 genes. Theoretically, our primers can recognize all of these splice variants but can not distinguish them in OLCs mRNA preparations. We also examed the presence of K_V_7.2-5 proteins in cultured OLCs. The signals of four proteins (K_V_7.2-5) were weakly detected in differentiated OLCs (IOs and MOs), while they were obviously dyed out in OPCs. In agreement with the immunostaining experiments, the inward deactivation relaxation currents almost did not exist in IOs and MOs. It is likely that the mRNAs for K_V_7.2-4, or only K_V_7.4 which were detected in IOs and MOs respectively were unable to be translated into enough Kv7 proteins to be detected by immunocytochemistry or electrophysiology method. This developmental regulation may reflect some yet unknown roles played by K_V_7 channels in early development of OLCs.

However, previous work on the expression of members of the Kv channel family suggested that the precise topographical distribution of Kv channel subunits in cultured cells may not fully reproduce that obtained in situ [64], [65]. Similarly, we detected the immunoreactive signal of K_V_7.2, 3 and 5 proteins, but not K_V_7.4, in NG2^+^ OPCs in rat cortex slices. These results agree with the work of Kharkovets et al. [22] that the cortex does not contain K_V_7.4 channel transcripts.

### The electrophysiological properties of K_V_7/KCNQ channels in OPCs

Functional K_V_7 channels are composed of four homomeric or heteromeric subunits. The sensitivity to XE991 of these homomeric or heteromeric channel currents differs considerably. Homomeric K_V_7.2 channels have an IC_50_ value for XE991 inhibition which is 0.7 µM. The K_V_7.2+3 heteromultimer retain the sensitivity of the K_V_7.2 homomultimer. However, homomeric K_V_7.3 and homomeric K_V_7.5 are very insensitive to XE991 with estimated IC_50_ values of <50 µM and 65 µM [2]. In our study, XE991 (10 µM) showed a inhibition of current (by 46.1±6.8%) and high concentration (30 µM) displayed over a half inhibition of I_K(Q)_ (by 67.4±5.6%) in OPCs. The IC50 for XE991 was 13.3 µM.

Different K_V_7 channel proteins also have different sensitivities to TEA [55]; [56]; [5]. In the present experiments, the I_K(Q)_ relaxations were completely inhibited by TEA with an IC50 of 0.84 mM, which is less than the IC50 for block of artificially expressed homomeric K_V_7.4 channels and heteromeric K_V_7.2/3 channels currents [5]; [55], though somewhat higher than the IC50 for block of homomeric K_V_7.2 currents. K_V_7.2-5 channel proteins were all detected in OPCs, however, their exactly expression level was unknown. The difference of pharmacological sensitivity to XE991 or TEA might due to various expression level and composition of each K_V_7 channel subunit in OPCs.

In the present study, the amplitude of I_K(Q)_ in OPCs was found to be voltage dependent, which is similar to I_M_ measured in sympathetic ganglion, hippocampal and dopamine neurons [3], [5], [8], [66], [67]. The maximal I_K(Q)_ amplitude in the OPCs was obtained at −40 mV with the deactivation protocol. The deactivation time constant was voltage dependent in OPCs, becoming shorter at more hyperpolarized membrane potentials, as has been observed for native I_M_ currents in neuronal cell types [3], [5], [10], [67]. The time course of I_K(Q)_ deactivation in OPCs was well fitted with a single exponential function and the value of the deactivation time constant was 152.85 ms at −50 mV. The deactivation time constant in OPCs seems to be closest to the fast component of the deactivation time constant in sympathetic neurons, which was reported to be 145 ms at −50 mV [5]. Native I_M_ currents in neurons have a biphasic (double-exponential) time course [5], [8], [10]. The absence of this slow component of deactivation in our experiments could be attributable to a difference in the types of K_V_7 channels underlying I_K(Q)_ in OPCs.

### Function of K_V_7/KCNQ in OPCs

During development, OLCs express all six members of the delayed rectifier Shaker family K^+^ channels, Kv1.1–Kv1.6 [40]–[45], inwardly rectifying K^+^ (K_ir_) channels Kir2.1, Kir1.1 and Kir4.1 [46], [47] and Kv3.1 [29]. In our experiments, we found that K_V_7 channels were expressed in OLCs, and downregulated in IOs and MOs. This developmental regulation may reflect some yet unknown roles played by K_V_7 channels in early OLCs development.

Migration of OPCs from proliferation zones to their final position is an essential step in the development of the nervous system [26], [68], [69], yet the physiological mechanisms of OPCs migration are still largely unknown. The idea that the K^+^ channels may be linked to cell migration is supported by several studies [36]–[39]. Importantly, K_V_7.1 potassium channels have been implicated recently in the regulation of migration and invasiveness of stem-like cell types [35]. Our results support the concept that K_V_7 channels are important for the regulation of OPCs migration in vitro. In our migration assay, the motility of the OPCs was promoted by the inhibition of K_V_7 channels. In fact, in neurons, K_V_7 channels can be inhibited by many endogenous factors. For example, stimulation of a variety of Gq/11-coupled neurotransmitter receptors, local changes in PIP2 concentration [70]–[72] and calmodulin [73], etc. In dissociated rat superior cervical sympathetic neurons, purinergic P2Y receptors can couple to G protein thereby modulating K_V_7 channel [74]. Agresti et al. [32] found that activation of P2Y1 receptors by ATP can promote OPCs migration. It is likely that ATP released following neuronal activity, astrocyte Ca^2+^ waves or cell lysis [75], [76] might inhibit K_V_7 channels though P2Y1, and consequently promote the OPCs migration.

## Materials and Methods

### Oligodendrocyte lineage cell cultures

The animal experiments were carried out in adherence with the National Institutes of Health Guidelines on the Use of laboratory Animals and were approved by Second Military Medical University Committee on Animal Care (permission <$>\raster="rg1"<$>: SCXK-HU-2007-0003). OLCs were prepared as previously described [77], [78] with slight modification. Briefly, cortex was dissected from postnatal day 1–2 Sprague Dawley rats, dissociated in Hanks balanced salt solution containing 0.125% trypsin (GIBCO, Canada) for 20 min, 37°C, suspended in DMEM containing 10% fetal bovine serum (FBS, BIOSOURCE, Brazil), and plated in plastic T75 flasks. After about 10 days in culture, OPCs growing on top of a confluent monolayer of astrocytes were detached by overnight shaking. Contaminating microglial cells were further eliminated by plating this fraction on plastic culture dishes for 1 hr. The OPCs, which do not attach well to plastic, were collected by gently washing the dishes, replated (3×10^4^ cells/cm^2^) onto poly-L-lysine-precoated plates (0.1 mg/ml) and cultured in DMEM containing 10% FBS medium. After 2 hr, DMEM supplemented with 30% B104 neuroblastoma conditioned medium, 1% B27 (GIBCO, Canada) and 1% N2 (GIBCO, Canada) was added to the culture medium. Immature oligodendrocytes (IOs) were produced by substitution of DMEM, 30% B104, 1% B27 and 1% N2 with Neurobasal medium (GIBCO, Canada) containing 1% B27, 1% N2, T3 (40 ng/ml) and biotin (10 ng/ml) for 2 days, and mature oligodendrocytes (MOs) by substitution with that medium for 4 days.

### RT-PCR and Quantitative PCR

Total RNA was extracted from adult rat forebrain or cultured OLCs using TRIZOL Reagent (Invitrogen Corporation, Carlsbad, CA), followed by the treatment with DNase I RNA-free (Fermentas, USA). Synthesis of cDNA was carried out with the Superscript First-Strand Synthesis System for RT-PCR (Invitrogen Corporation, Carlsbad, CA). The RNA from OLCs without reverse transcription was performed PCR procedure directly as negative control, while the RNA from adult rat forebrain was as positive control. The PCR temperature profile was 94°C for 3 min, followed by 40 cycles of 94°C for 30 s, 60°C for 30 s, and 72°C for 1 min, 72°C 5 min. All primers are listed in Table 1.

**Table 1:** Primers for PCR.

Gene	Accession no.	Primer(5′–3′)	Product size(bp)
KCNQ2	NM133322	F: GGTGCTGATTGCCTCCATTR: CTCCTTGCTGTGAGCGTAGAC	172 bp
KCNQ3	NM031597	F: CCCCTATTCGGACCACATCR: GCTGAAGCCACTTGGAGACC	121 bp
KCNQ4	XM233477	F: GACGATTACACTGACGACCATTR: GCAGGGCAAAGAAGGAGAT	110 bp
KCNQ5	XM001071249	F: GCTGGGCTCCGTGGTTTAR: TCTGGCGGTGCTGTTCCT	320 bp
GAPDH	NM017008	F:TCTGACATGCCGCCTGGAGAAACCTGCR:CACCACCCTGTTGCTGTAGCCATATTCATTGTC	243 bp

Specifies forward (F) and reverse (R) primers used for RT-PCR of rat KCNQ channel subunits and GAPDH.

### Immunocytochemistry and immunohistochemistry

For immunocytochemical analysis, OLCs on coverslips were washed with phosphate buffered saline (PBS) and fixed with 4% paraformaldehyde (PFA) in PBS for 20 min, followed by permeabilization with 0.3% Triton X-100 in 0.1 M PBS for 10 minutes. After blocking the non-specific binding with 10% normal goat serum or 1% BSA in 0.1 M PBS, cells were incubated with primary antibodies against A2B5 (Chemicon, USA), GFAP (Sigma, USA), NG2 (Chemicon, USA), O4 (Sigma, USA), MBP (Chemicon,USA), K_V_7.2, 3 (Chemicon, USA) , K_V_7.4 (Santa Cruz, USA) and K_V_7.5 (Millipore, USA) at 4°C overnight. Cells were then washed and incubated with fluorescence-conjugated secondary antibodies (Jackson ImmunoResearch Laboratories, Inc.) for 6 hours at room temperature and examined by fluorescence microscopy (Nikon, Japan). For immunohistochemical analysis, animals were deeply anaesthetized with 2% pentobarbital sodium and perfused transcardially with 4% PFA in 0.1 M PBS, pH 7.4. The brain were subsequently dissected from each animal and post-fixed in the perfusing solution overnight at 4°C. Then, the tissues were cryoprotected in 20% sucrose in PBS for 24–48 h at 4°C. Cryostat sections (10 µm) were cut and mounted onto gelatin-subbed slides and stored at −20°C. For immunostaining, the protocol performed was similar to immunocytochemical analysis.

### Electrophysiological recordings

Current recordings were performed in the whole cell configuration of the patch-clamp technique using MultiClamp700A amplifier (Axon, USA). Date were stored in a PC, and analyzed by pClamp8.02 software (Axon Instruments, Sunnyvale, CA, USA). The patch pipettes (6∼8 MΩ), pulled using a Narishige puller (PP-83, Japan) and polished using a MF200 Microforge (WPI, USA), were filled with solution containing 140 mM KCl, 4 mM MgCl_2_, 0.1 mM EGTA, 4 mMATP·2Na, 0.5 mM Na_3_·GTP, and 10 mM HEPES (pH 7.4 with KOH). The superfusate solution used to measure I_K(Q)_ contained 150 mM NaCl, 5 mM KCl, 1 mM MgCl_2_, 2 mMCaCl_2_, 10 mM HEPES, and 10 mM Glucose (pH 7.4 with NaOH). All experiments were done in room temperature. Drugs were applied through OctaFlow System (ALA, USA) to the cell under recording. XE991 (Sigma, USA) and TEA (Sigma, USA) was dissolved in water to store at −20°C, and diluted part per thousand using superfusate solution. Hyperpolarizing voltage steps (1 s duration) were given from a holding potential of −20 to −60 mV (in 10-mV increments).

Graphing and curve fitting of data were performed with Origin 7 software (OriginLab, Northampton, MA). The inward relaxation current, which was attributed to deactivation of I_K(Q)_, was fitted by a single exponential function 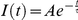. Where A is amplitude obtained from the beginning of the fit and τ is the decay time constant.

Concentration-response curves for XE991 and TEA were constructed by plotting percentage inhibition of I_K(Q)_ as a function of drug concentration plotted on a log scale. Smooth curves were fit to these data with the Hill equation 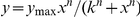.

Where x is the concentration, y is the percentage inhibition, and y_max_ is the maximal value of y (at saturation); in the fitting procedure y_max_ was constrained not to exceed 100%. The term k is the IC50 (the concentration giving half-maximal inhibition) and n (Hill slope) is the power term related to the slope of the curve.

### Boyden chamber migration assay

To measure the motility of OPCs, Boyden chamber migration assay was performed as previously described [79]. In brief, the polyethylene terephthalate filter membranes were coated with poly-L-lysine. The purified OPCs were seeded onto the upper chamber at a density of 2×10^5^ cells in 200 µl of culture medium containing 10% FBS per well, and 600 uL DMEM containing 10% FBS were added to lower chamber. When OPCs were adherent (about 40 min later), the DMEM medium containing 10% FBS in the upper and lower chamber was replaced with serum-free DMEM supplemented with 30% B104 neuroblastoma conditioned medium, 1%B27, and 1%N2. XE991 or TEA was added to the lower chamber. After incubation for 8 h at 37°C, non-migratory cells on the upper membrane surface were removed with a cotton swab, and migratory cells invading to the underside surface of the membrane were fixed with 4% paraformaldehyde and stained with Coomassie Brilliant Blue. For quantitative assessment, the number of stained cells was counted under microscopy at 12 fields per filter in three independent experiments.

### Statistical analysis

Data from at least three independent experiments were all presented as means ± SEM. Statistical significance was evaluated with paired Student's t-test. Differences were considered significant at p<0.05.

## References

[1] Jentsch TJ (2000). Neuronal KCNQ potassium channels: physiology and role in disease.. Nat Rev Neurosci.

[2] Robbins J (2001). KCNQ potassium channels: physiology, pathophysiology, and pharmacology.. Pharmacol Ther.

[3] Brown DA, Adams PR (1980). Muscarinic suppression of a novel voltage-sensitive K^+^ current in a vertebrate neurone.. Nature.

[4] Brown DA (1988). M currents.. Ion Channels.

[5] Wang HS, Pan Z, Shi W, Brown BS, Wymore RS (1998). KCNQ2 and KCNQ3 potassium channel subunits: molecular correlates of the M-channel.. Science.

[6] Hadley JK, Passmore GM, Tatulian L, Al-Qatari M, Ye F (2003). Stoichiometry of expressed KCNQ2/KCNQ3 channels and subunit composition of native ganglionic M-channels deduced from block by tetraethyl ammonium (TEA).. J Neurosci.

[7] Schwarz JR, Glassmeier G, Cooper E, Kao T, Nodera H (2006). KCNQ channels mediate IKs, a slow K^+^ current regulating excitability in the node of Ranvier.. J Physiol.

[8] Shah MM, Mistry M, Marsh SJ, Brown DA, Delmas P (2002). Molecular correlates of the M-current in cultured rat hippocampal neurons.. J Physiol.

[9] Hansen HH, Waroux O, Seutin V, Jentsch TJ, Aznar S (2008). Kv7 channels: interaction with dopaminergic and serotonergic neurotransmission in the CNS.. J Physiol.

[10] Passmore GM, Selyanko AA, Mistry M, Al-Qatari M, Marsh SJ (2003). KCNQ/M currents in sensory neurons: significance for pain therapy.. J Neurosci.

[11] Zaika O, Lara LS, Gamper N, Hilgemann DW, Jaffe DB (2006). Angiotensin-II regulates neuronal excitability via PIP2-dependent modulation of Kv7 (M-type) potassium channels.. J Physiol.

[12] Lang PM, Fleckenstein J, Passmore GM, Brown DA, Grafe P (2008). Retigabine reduces the excitability of unmyelinated peripheral human axons.. Neuropharmacology.

[13] Wladyka CL, Feng B, Glazebrook PA, Schild JH, Kunze DL (2008). The KCNQ/M-current modulates arterial baroreceptor function at the sensory terminal in rats.. J Physiol.

[14] Yue C, Yaari Y (2004). KCNQ/M channels control spike afterdepolarization and burst generation in hippocampal neurons.. J Neurosci.

[15] Liu B, Linley JE, Du X, Zhang X, Ooi L (2010). The acute nociceptive signals induced by bradykinin in rat sensory neurons are mediated by inhibition of M-type K^+^ channels and activation of Ca2^+^-activated Cl^−^ channels.. J Clin Invest.

[16] Aiken SP, Lampe BJ, Murphy PA, Brown BS (1995). Reduction of spike frequency adaptation and blockade of M-current in rat CA1 pyramidal neurones by linopirdine (DuP 996), a neurotransmitter release enhancer.. Br J Pharmacol.

[17] Peters HC, Hu H, Pongs O, Storm JF, Isbrandt D (2005). Conditional transgenic suppression of M channels in mouse brain reveals functions in neuronal excitability, resonance and behavior.. Nat Neurosci.

[18] Biervert C, Schroeder BC, Kubisch C, Berkovic SF, Propping P (1998). A potassium channel mutation in neonatal human epilepsy.. Science.

[19] Schroeder BC, Hechenberger M, Weinreich F, Kubisch C, Jentsch TJ (2000). KCNQ5, a novel potassium channel broadly expressed in brain, mediates M-type currents.. J Biological Chemistry.

[20] Kananura C, Biervert C, Hechenberger M, Engels H, Steinlein OK (2000). The new voltage gated potassium channel KCNQ5 and neonatal convulsions.. Neuroreport.

[21] Kubisch C, Schroeder BC, Friedrich T, Lütjohann B, El-Amraoui A (1999). KCNQ4, a novel potassium channel expressed in sensory outer hair cells, is mutated in dominant deafness.. Cell.

[22] Kharkovets T, Hardelin JP, Safieddine S, Schweizer M, El-Amraoui A (2000). KCNQ4, a K^+^ channel mutated in a form of dominant deafness, is expressed in the inner ear and the central auditory pathway.. Proc Natl Acad Sci U S A.

[23] Kharkovets T, Dedek K, Maier H, Schweizer M, Khimich D (2006). Mice with altered KCNQ4 K^+^ channels implicate sensory outer hair cells in human progressive deafness.. EMBO J.

[24] Richardson WD, Kessaris N, Pringle N (2006). Oligodendrocyte wars.. Nat Rev Neurosci.

[25] Bradl M, Lassmann H (2010). Oligodendrocytes: biology and pathology.. Acta Neuropathol.

[26] Kessaris N, Fogarty M, Iannarelli P, Grist M, Wegner M (2006). Competing waves of oligodendrocytes in the forebrain and postnatal elimination of an embryonic lineage.. Nat Neurosci.

[27] McTigue, DM, Tripathi, RB (2008). The life, death, and replacement of oligodendrocytes in the adult CNS.. J Neurochem.

[28] Blakemore WF, Keirstead HS (1999). The origin of remyelinating cells in the central nervous system.. J Neuroimmunol.

[29] Tiwari-Woodruff S, Beltran-Parrazal L, Charles A, Keck T, Vu T (2006). K^+^ channel K_V_3.1 associates with OSP/claudin-11 and regulates oligodendrocyte development.. Am J Physiol Cell Physiol.

[30] Paez PM, Fulton DJ, Spreuer V, Handley V, Campagnoni CW (2009). Golli myelin basic proteins regulate oligodendroglial progenitor cell migration through voltage-gated Ca^2+^ influx.. J Neurosci.

[31] Paez PM, Fulton DJ, Spreur V, Handley V, Campagnoni AT (2010). Multiple kinase pathways regulate voltage-dependent Ca^2+^ influx and migration in oligodendrocyte precursor cells.. J Neurosci.

[32] Agresti C, Meomartini ME, Amadio S, Ambrosini E, Volonté C (2005). ATP regulates oligodendrocyte progenitor migration, proliferation, and differentiation: involvement of metabotropic P2 receptors.. Brain Res Brain Res Rev.

[33] Luyt K, Slade TP, Dorward JJ, Durant CF, Wu Y (2007). Developing oligodendrocytes express functional GABA (B) receptors that stimulate cell proliferation and migration.. J Neurochem.

[34] Gudz TI, Komuro H, Macklin WB (2006). Glutamate stimulates oligodendrocyte progenitor migration mediated via an alphav integrin/myelin proteolipid protein complex.. J Neurosci.

[35] Morokuma J, Blackiston D, Levin M (2008). KCNQ1 and KCNE1 K^+^ channel components are involved in early left-right patterning in Xenopus laevis embryos.. Cell Physiol Biochem.

[36] Rao JN, Platoshyn O, Li L, Guo X, Golovina VA (2002). Activation of K^+^ channels and increased migration of differentiated intestinal epithelial cells after wounding.. Am J Physiol Cell Physiol.

[37] Toral C, Mendoza-Garrido ME, Azorín E, Hernández-Gallegos E, Gomora JC (2007). Effect of extracellular matrix on adhesion, viability, actin cytoskeleton and K^+^ currents of cells expressing human ether à go-go channels.. Life Sci.

[38] Lastraioli E, Guasti L, Crociani O, Polvani S, Hofmann G (2004). herg1 gene and HERG1 protein are overexpressed in colorectal cancers and regulate cell invasion of tumor cells.. Cancer Res.

[39] Matheu MP, Beeton C, Garcia A, Chi V, Rangaraju S (2008). Imaging of effector memory T cells during a delayed-type hypersensitivity reaction and suppression by Kv1.3 channel block.. Immunity.

[40] Bacia A, Wollmann R, Soliven B (2004). K^+^ channel blockade impairs remyelination in the cuprizone model.. Glia.

[41] Chittajallu R, Chen Y, Wang H, Yuan X, Ghiani CA (2002). Regulation of Kv1 subunit expression in oligodendrocyte progenitor cells and their role in G1/S phase progression of the cell cycle.. Proc Natl Acad Sci U S A.

[42] Gallo V, Zhou JM, McBain CJ, Wright P, Knutson PL (1996). Oligodendrocyte progenitor cell proliferation and lineage progression are regulated by glutamate receptor-mediated K^+^ channel block.. J Neurosci.

[43] Knutson P, Ghiani CA, Zhou JM, Gallo V, McBain CJ (1997). K^+^ channel expression and cell proliferation are regulated by intracellular sodium and membrane depolarization in oligodendrocyte progenitor cells.. J Neurosci.

[44] Shrager P, Novakovic SD (1995). Control of myelination, axonal growth, and synapse formation in spinal cord explants by ion channels and electrical activity.. Brain Res Dev Brain Res.

[45] Attali B, Wang N, Kolot A, Sobko A, Cherepanova V (1997). Characterization of delayed rectifier Kv channels in oligodendrocytes and progenitor cells.. J Neurosci.

[46] Karschin A, Wischmeyer E (1995). Identification of G protein-regulated inwardly rectifying K^+^ channels in rat brain oligodendrocytes.. Neurosci Lett.

[47] Neusch C, Rozengurt N, Jacobs RE, Lester HA, Kofuji P (2001). Kir4.1 potassium channel subunit is crucial for oligodendrocyte development and in vivo myelination.. J Neurosci.

[48] Dubois-Dalcq M (1987). Characterization of a slowly proliferative cell along the oligodendrocyte differentiation pathway.. EMBO J.

[49] Gard AL, Pfeiffer SE (1989). Oligodendrocyte progenitors isolated directly from developing telencephalon at a specific phenotypic stage: myelinogenic potential in a defined environment.. Dev Biol.

[50] Adams PR, Brown DA (1982). Synaptic inhibition of the M-current: slow excitatory post-synaptic potential mechanism in bullfrog sympathetic neurones.. J Physiol (Lond).

[51] Nisenbaum ES, Wilson CJ, Foehring RC, Surmeier DJ (1996). Isolation and characterization of a persistent potassium current in neostriatal neurons.. J Neurophysiol.

[52] Tkatch T, Baranauskas G, Surmeier DJ (2000). Kv4.2 mRNA abundance and A-type K^+^ current amplitude are linearly related in basal ganglia and basal forebrain neurons.. J Neurosci.

[53] Shen W, Hernandez-Lopez S, Tkatch T, Held JE, Surmeier DJ (2004). Kv1.2-containing K^+^ channels regulate subthreshold excitability of striatal medium spiny neurons.. J Neurophysiol.

[54] Wladyka CL, Kunze DL (2006). KCNQ/M-currents contribute to the resting membrane potential in rat visceral sensory neurons.. J Physiol.

[55] Hadley JK, Noda M, Selyanko AA, Wood IC, Abogadie FC (2000). Differential tetraethylammonium sensitivity of KCNQ1-4 potassium channels.. Br J Pharmacol.

[56] Shapiro MS, Roche JP, Kaftan EJ, Cruzblanca H, Mackie K (2000). Reconstitution of muscarinic modulation of the KCNQ2/KCNQ3 K^+^ channels that underlie the neuronal M current.. J Neurosci.

[57] Cooper EC, Harrington E, Jan YN, Jan LY (2001). M channel KCNQ2 subunits are localized to key sites for control of neuronal network oscillations and synchronization in mouse brain.. J Neurosci.

[58] Saganich MJ, Machado E, Rudy B (2001). Differential expression of genes encoding subthreshold-operating voltage-gated K^+^ channels in brain.. J Neurosci.

[59] Yus-Nájera E, Muñoz A, Salvador N, Jensen BS, Rasmussen HB (2003). Localization of KCNQ5 in the normal and epileptic human temporal neocortex and hippocampal formation.. Neuroscience.

[60] Tinel N, Lauritzen I, Chouabe C, Lazdunski M, Borsotto M (1998). The KCNQ2 potassium channel: splice variants, functional and developmental expression. Brain localization and comparison with Kcnq3.. FEBS Lett.

[61] Jeffrey SS, Claudia AI, Pauline D, Edward PC, Jayashree A (2001). Differential Expression of KCNQ2 Splice Variants: Implications to M Current Function during Neuronal Development.. J Neurosci.

[62] BjÖrn CS, Mirko H, Frank W, Christian K, Thomas JJ (2000). KCNQ5, a Novel Potassium Channel Broadly Expressed in Brain, Mediates M-type Currents.. J Biological chemistry.

[63] Kirk WB, Sonia MRS, Ken AM, Liping N, Feng F (2005). Differential Expression of KCNQ4 in Inner Hair Cells and Sensory Neurons Is the Basis of Progressive High-Frequency Hearing Loss.. J Neurosci.

[64] Maletic-Savatic M, Lenn NJ, Trimmer JS (1998). Differential spatiotemporal expression of K^+^ channel polypeptides in rat hippocampal neurons developing in situ and in vitro.. J Neurosci.

[65] Grosse G, Draguhn A, Höhne L, Tapp R, Veh RW (2000). Expression of Kv1 potassium channels in mouse hippocampal primary cultures: development and activity-dependent regulation.. J Neurosci.

[66] Schweitzer P (2000). Cannabinoids decrease the K^+^ M-current in hippocampal CA1 neurons.. J Neurosci.

[67] Koyama S, Appel SB (2006). Characterization of M-current in ventral tegmental area dopamine neurons.. J Neurophysiol.

[68] Ivanova A, Nakahira E, Kagawa T, Oba A, Wada T (2003). Evidence for a second wave of oligodendrogenesis in the postnatal cerebral cortex of the mouse.. J Neurosci Res.

[69] Baumann N, Pham-Dinh D (2001). Biology of oligodendrocyte and myelin in the mammalian central nervous system.. Physiol Rev.

[70] Zhang H, Craciun LC, Mirshahi T, Rohács T, Lopes CM (2003). PIP(2) activates KCNQ channels, and its hydrolysis underlies receptor-mediated inhibition of M currents.. Neuron.

[71] Delmas P, Brown DA (2005). Pathways modulating neural KCNQ/M (Kv7) potassium channels.. Nat Rev Neurosci.

[72] Brown DA, Passmore GM (2009). Neural KCNQ (Kv7) channels.. Br J Pharmacol.

[73] Gamper N, Shapiro MS (2003). Calmodulin mediates Ca2^+^-dependent modulation of M-type K^+^ channels.. J Gen Physiol.

[74] Brown DA, Filippov AK, Barnard EA (2000). Inhibition of potassium and calcium currents in neurones by molecularly-defined P2Y receptors.. J Auton Nerv Syst.

[75] Pankratov Y, Lalo U, Verkhratsky A, North RA (2006). Vesicular release of ATP at central synapses.. Pflugers Arch.

[76] Schipke CG, Boucsein C, Ohlemeyer C, Kirchhoff F, Kettenmann H (2002). Astrocyte Ca^2+^ waves trigger responses in microglial cells in brain slices.. FASEB J.

[77] Armstrong RC, Harvath L, Dubois-Dalcq M (1990). Type 1 astrocytes and oligodendrocytes-type 2 astrocytes glial progenitors migrate toward distinct molecules.. J Neurosci Res.

[78] McKinnon RD, Matsui T, Dubois-Dalcq M, Aaronson SA (1990). FGF modulates the PDGF-driven pathway of oligodendrocyte development.. Neuron.

[79] Cao L, Su ZD, Zhou Q, Lv BL, Liu XJ (2006). Glial cell line-derived neurotrophic factor promotes olfactory ensheathing cells migration.. Glia.

